# Modified Particle Filtering Algorithm for Single Acoustic Vector Sensor DOA Tracking

**DOI:** 10.3390/s151026198

**Published:** 2015-10-16

**Authors:** Xinbo Li, Haixin Sun, Liangxu Jiang, Yaowu Shi, Yue Wu

**Affiliations:** 1 School of Communication Engineering, Jilin University, Renmin Street No. 5988, Changchun 130022, China; E-Mails: cinple@126.com (X.L.); jiangalbert@126.com (L.J.); syw@jlu.edu.cn (Y.S.); 2 School of Electronic and Information Engineering, Changchun University, Weixing Road, No. 6543, Changchun 130022, China; E-Mail: haixin_s@hotmail.com; 3 School of Mechanical Science and Engineering, Jilin University, Renmin Street No. 5988, Changchun 130022, China

**Keywords:** DOA tracking, particle filtering, importance function, acoustic vector sensor

## Abstract

The conventional direction of arrival (DOA) estimation algorithm with static sources assumption usually estimates the source angles of two adjacent moments independently and the correlation of the moments is not considered. In this article, we focus on the DOA estimation of moving sources and a modified particle filtering (MPF) algorithm is proposed with state space model of single acoustic vector sensor. Although the particle filtering (PF) algorithm has been introduced for acoustic vector sensor applications, it is not suitable for the case that one dimension angle of source is estimated with large deviation, the two dimension angles (pitch angle and azimuth angle) cannot be simultaneously employed to update the state through resampling processing of PF algorithm. To solve the problems mentioned above, the MPF algorithm is proposed in which the state estimation of previous moment is introduced to the particle sampling of present moment to improve the importance function. Moreover, the independent relationship of pitch angle and azimuth angle is considered and the two dimension angles are sampled and evaluated, respectively. Then, the MUSIC spectrum function is used as the “likehood” function of the MPF algorithm, and the modified PF-MUSIC (MPF-MUSIC) algorithm is proposed to improve the root mean square error (RMSE) and the probability of convergence. The theoretical analysis and the simulation results validate the effectiveness and feasibility of the two proposed algorithms.

## 1. Introduction

Acoustic vector sensor is a new kind of sensors that can obtain the information of the scalar field and the vector field of acoustic wave simultaneously. Its application covers the areas of mobile positioning, sonar system, radar tracking, fault source location, *etc*. Since single acoustic vector sensor is a simplified version of the acoustic vector array and its array aperture can be expanded for multiple sources location estimation, some related issues have been considered [1,2,3,4,5,6,7]. The near-field manifold of single acoustic vector sensor has been derived in [1]. Then, the azimuth-elevation direction of arrival plus radial distance estimation algorithm for near-field wideband emitter was developed in [2]. The beamforming and blind interference rejection algorithm for fast frequency-hop spread spectrum signals was presented in [3]. The Cramer-Rao bound for single acoustic vector sensor DOA estimation with model errors was investigated in [4,5]. The spatial collocation requirement of single acoustic vector sensor was relaxed and four-component sensors were located separately, which realizes an extended spatial aperture [6,7]. Usually, the DOA estimation algorithm based on acoustic vector sensor adopts multiple snapshot and batch processing to estimate the target parameters, which is also named as static DOA estimation [1,2,3,4,5,6,7,8,9,10]. However, in the real environment, the target source is moving and the batch processing of the static DOA estimation algorithm cannot achieve the continuous tracking. Furthermore, the static estimation algorithm estimates the angles of two different moments independently and does not consider the correlation of the moments, which leads to the large dynamic estimation deviation. The conventional static DOA estimation algorithm cannot fulfill the increasing accuracy and real-time demands. Hence, the study of DOA tracking estimation for dynamic sources becomes a new trend. 

To handle the DOA tracking for dynamic sources, several non-stationary adaptive algorithms have been proposed, such as matched-field processing [11], H_∞_ tracking [12], Kalman filtering [13], adaptive recursive-least-squares tracking [14,15] particle filtering (PF) [16,17,18,19,20], *etc.* The objectives of these algorithms are to improve the dynamic localization performance. Since particle filtering can deal with any dynamic system of linear Gaussian or nonlinear non-Gaussian represented by state space models [21,22,23,24,25,26] and the observation equation of the acoustic vector sensor is a highly nonlinear function of state, the PF algorithm has been introduced to the field of the acoustic vector array signal processing. In [17], the single acoustic vector sensor DOA tracking algorithm was proposed with particle filtering, the maximum “likehood” function was used as the observation likehood function and the Cramer-Rao bound for dynamic DOA estimation under the Gaussian background was derived in details. In [18], the CAPON and Barlett beamforming spectrum functions were used as the likehood function of the PF algorithm and the modified particle filtering DOA tracking algorithm with the exponential factor weighting was proposed. In [19], the algorithms [17,18] were adopted and verified in the real marine environment and the effectiveness and anti-jamming capability of different algorithms were validated. The final results indicated that the algorithm proposed in [18] outperformed other algorithms in the real marine noise environment. The algorithms [17,18] were established under the conditions of Gaussian background noise. In [20], the DOA tracking under the impulsive noise was studied. The observation likehood function based on fractional order covariance matrix was derived and the combination of the fractional low order of moment and the PF (FLOM-PF) algorithm was proposed. 

The study results mentioned above presented that the CAPON algorithm is affected by the number of array elements. When the element number is small, the estimation deviation is large. However, the MUSIC algorithm had better resolution performance and sharper spectrum peak when the distance of the targets is close [27]. The MUSIC algorithms for acoustic vector sensor applications have been presented in [28,29,30]. As the likehood function of the particle filtering, the spectrum function of the MUSIC algorithm has better evaluation for the particles instead of the spectrum functions of CAPON algorithm or Barlett beamforming algorithm. 

On the basis of previous studies, the contents of this paper are as follows. Firstly, considering the independent relationship of pitch angle and azimuth angle, the two angles are sampled and evaluated independently to avoid large deviation of either pitch angle or azimuth angle, which leads to the other angle being unable to update the state accurately, through resampling, and a modified PF algorithm is proposed which improves the importance probability density. Secondly, the spectrum function of MUSIC algorithm is exploited as the likehood function of PF, instead of the spectrum functions of CAPON algorithm and Barlett beamforming algorithm, and the modified PF-MUSIC tracking algorithm is proposed. Finally, the feasibility and effectiveness of the algorithms are validated through simulation results.

## 2. State Space Model

### 2.1. The Establishment of the State Equation

Since considering multiple acoustic sources existing simultaneously is more complex than a single acoustic source, we mainly consider the single acoustic source issue. Assuming the moving speed of DOA is constant, the variation of the source angle is simulated by the two order constant velocity model and the state equation of single angle is established. The case of multiple state variables can be derived in a similar way. 

Given that the source angle is represented by *θ* moving along a straight line in a certain period of time and the sampling time is ΔT, at the sampling point *k*ΔT, the real pitch angle of the target *θ*(*k*), the moving speed is $\overset{\cdot }{\theta }\left(k\right)$. According to the equation of the constant velocity motion, we have

(1) $$\theta \left(k+1\right)=\theta \left(k\right)+\overset{\cdot }{\theta }\left(k\right)\Delta T+\frac{1}{2}\Delta T^{2}u\left(k\right)$$

(2) $$\overset{\cdot }{\theta }\left(k+1\right)=\overset{\cdot }{\theta }\left(k\right)+\Delta Tu\left(k\right)$$

where *u*(*k*) is the speed fluctuations caused by external factors such as friction force or wind [19]. Assuming that the noise is zero-mean Gaussian random noise and independent with the observation noise, the state space model of variable *θ* can be derived as,

(3) $$\left[\begin{array}{c} \theta \left(k+1\right)\\ \overset{\cdot }{\theta }\left(k+1\right) \end{array}\right]=\left[\begin{array}{cc} 1 & T_{0}\\ 0 & 1 \end{array}\right]\left[\begin{array}{c} \theta \left(k\right)\\ \overset{\cdot }{\theta }\left(k\right) \end{array}\right]+\left[\begin{array}{c} \frac{1}{2}\Delta T^{2}\\ \Delta T \end{array}\right]u\left(k\right)$$

Similarly, consider the case of the source state expanded to two angles. In the k moment, the four-dimensional state vector of the source is represented by $x_{k}={\left[\theta_{k},\overset{\cdot }{\theta_{k}},\varphi_{k},\overset{\cdot }{\varphi_{k}}\right]}^{T}$, where *θ_k_* is the pitch angle, ${\overset{\cdot }{\theta }}_{k}$ is the velocity variable of pitch angle, *φ_k_* is the azimuth angle and ${\overset{\cdot }{\varphi }}_{k}$ is the velocity variable of azimuth angle. Hence, the state equation of the acoustic source angle is

(4) $$x_{k}=Ax_{k-1}+Bu_{k}$$

where *u_k_* is zero-mean Gaussian random noise, *A* is the state coefficient matrix and *B* is the input coefficient matrix. For the DOA estimation and tracking of the acoustic vector sensor, the value of *A* and *B* are

(5) $$A=\left[\begin{array}{l} 1,\Delta T,0,0\\ 0,\;1,\;0,0\\ 0,0,1,\Delta T\\ 0,0,\;0,\;1 \end{array}\right],\;B=\left[\begin{array}{c} \Delta T^{2}/2\\ \Delta T\\ \Delta T^{2}/2\\ \Delta T \end{array}\right]$$

### 2.2. The Establishment of the Observation Model

Assuming a far-field narrowband signal with *λ_s_* wavelength impinges into a single acoustic vector sensor with impinging angles $\Theta_{s}=\left(\theta_{s},\varphi_{s}\right)$, where $\varphi_{s}\in (0,2\pi )$ is the pitch angle and $\theta_{s}\in (-\pi /2,\pi /2)$ is the azimuth angle of the impinging signal, respectively. The sensor location in the coordinates is $r=(x_{0},y_{0},z_{0})$. The sound pressure of the source is shown as *s*(*t*), which is an unknown deterministic signal. The unit vector ${\vec{u}}_{s}$ is $(\sin\theta_{s}\cos\varphi_{s},\sin\theta_{s}\sin\varphi_{s},$ $\cos\theta_{s})$, which is pointed from the origin to the direction of the acoustic source. The vibration velocities of the acoustic source on the x–y–z coordinate axes are represented by $v_{x}(t),v_{y}(t),v_{z}(t)$, respectively. According to the relationship of the particle vibration velocities and the sound pressure, we have:

(6) $$\begin{array}{l} v_{x}(t)=\sin\theta_{s}\cos\varphi_{s}*s(t)\\ v_{y}(t)=\sin\varphi_{s}\sin\theta_{s}*s(t)\\ v_{z}(t)=\cos\theta_{s}*s(t) \end{array}$$

The wave delay of the acoustic source in the present sensor element is

(7) $$\Phi_{s}=\frac{2\pi }{\lambda }(x_{0}\cos\theta_{s}\sin\varphi_{s}+y_{0}\sin\theta_{s}\sin\varphi_{s}+z_{0}\cos\varphi_{s})$$

The output of the array element is represented by

(8) $$y(t)=a(\Phi_{s})⊗{\left[1,\cos\theta_{s}\sin\varphi_{s},\sin\theta_{s}\sin\varphi_{s},\cos\varphi_{s}\right]}^{T}s(t)+n(t)$$

where $y(t)\subset C^{4*1}$, ${\left[\cdot \right]}^{T}$represents the transpose operation, ⊗ represents the direct product, *n*(*t*) is the interference noise at the receiving end, and $a(\Phi_{s})$ is the sensor response coefficient to the acoustic source, and it is:

(9) $$a(\Phi_{s})=\exp\left(-j\Phi_{s}\right)$$

In the practical engineering application, to guarantee the real-time and effectiveness of the estimation, the observation matrix of the array is obtained with the limited number of snapshots. Assuming *L* observations at the *k* moment, the receiving matrix is:

(10) $$\begin{array}{c} Y\left(k\right)=a(\Phi_{s}){\left[1,\cos\theta_{s}\sin\varphi_{s},\sin\theta_{s}\sin\varphi_{s},\cos\varphi_{s}\right]}^{T}⊗s(k)+\text{N}(k)\\ =b(\Phi_{s})⊗s(k)+\text{N}(k) \end{array}$$

where

(11) $$b(\Phi_{s})=a(\Phi_{s}){\left[1,\cos\theta_{s}\sin\varphi_{s},\sin\theta_{s}\sin\varphi_{s},\cos\varphi_{s}\right]}^{T}$$

(12) s(k)=[s(kL+1),s(kL+2),⋅⋅⋅,s(kL+L)]

## 3. Modified Particle Filtering Tracking Algorithm

### 3.1. Particle Filtering Algorithm

The Bayes importance sampling algorithm [19] can be achieved with sequential importance sampling (SIR) process in recursive way and the state filtering estimation can be accomplished in predicting and updating way. The prediction process is

(13) $$p\left(x_{k}|Y_{1:k-1}\right)=\int p\left(x_{k}|x_{k-1}\right)p\left(x_{k-1}|Y_{1:k-1}\right)dx_{k-1}$$

where p(·) represents the conditional probability. The update process is

(14) $$p\left(x_{k}|Y_{1:k}\right)=\frac{p\left(Y_{k}|x_{k}\right)p\left(x_{k}|Y_{1:k-1}\right)}{\int p\left(Y_{k}|x_{k}\right)p\left(x_{k}|Y_{1:k-1}\right)dx_{k}}$$

Considering the relationship of the two adjacent observing time, the sequential importance sampling process calculates the sampling weights through recursion. At the k moment, the updated equation of the weight of the particle *x_k_* (*i*) is

(15) $$w_{k}^{*}\left(x_{k}\left(i\right)\right)=w_{k-1}^{*}\left(x_{k-1}\left(i\right)\right)\frac{p\left(Y_{k}|x_{k}\left(i\right)\right)p\left(x_{k}\left(i\right)|x_{k-1}\left(i\right)\right)}{q\left(x_{k}\left(i\right)|x_{0:k-1}\left(i\right),Y_{1:k}\right)}$$

where $q\left(x_{k}|x_{0:k-1},Y_{1:k}\right)$ is the importance probability density, $x_{k}\left(i\right),i=1,\cdot \cdot \cdot ,N$ is the particle samples at the k moment. In the SIR algorithm [23], the simplified $p\left(x_{k}|x_{k-1}\right)$ is employed as the importance probability density and the particle samples are extracted randomly from this distribution, which is represented by

(16) $$q\left(x_{k}\left(i\right)|x_{0:k-1}\left(i\right),Y_{1:k}\right)=p\left(x_{k}\left(i\right)|x_{k-1}\left(i\right)\right)$$

The corresponding weight calculating formula of the particles is

(17) $$w_{k}^{*}\left(x_{k}\left(i\right)\right)=w_{k-1}^{*}\left(x_{k-1}\left(i\right)\right)p\left(Y_{k}|x_{k}\left(i\right)\right)$$

The normalized weight coefficient is

(18) $$w_{k}\left(x_{k}\left(i\right)\right)=\frac{w_{k}^{*}\left(x_{k}\left(i\right)\right)}{\sum_{i=1}^{N}w_{k}^{*}\left(x_{k}\left(i\right)\right)}$$

Hence, the estimated value of *x_k_* is

(19) $$x_{k}=\sum_{i=1}^{N}w_{k}\left(i\right)x_{k}\left(i\right)$$

### 3.2. Modified Particle Filtering Algorithm

The system state includes two parameters, the pitch angle and the azimuth angle of the impinging signal. From the derivation of the particle filtering algorithm [19], it is known that (*θ_k_* (*i*), *φ_k_* (*i*)) is used as a state combination for estimation. Hence, the algorithms in [19] may encounter some problems, especially when the pitch angle is in the vicinity of the real state while the azimuth angle has large deviation from the real state, or *vice versa*, the weight *w_k_* (*i*) for the combination of angles (*θ_k_* (*i*), *φ_k_* (*i*)) is small relatively. The small weight samples cannot be duplicated and propagated by resampling process. Thus, the effective pitch angle (or azimuth angle) samples in the vicinity of the real state are overlooked due to not passing the resampling process. Hence, in the case of two estimated angles, the two angles should be evaluated independently. The problems that the expected angles cannot be duplicated and propagated through resampling process with large deviation of pitch angle or azimuth angle can be avoided.

Here, the improvements of the evaluation way for the pitch angle and the azimuth angle are introduced to the SIR particle filtering. The corresponding importance probability density functions of the pitch angle and the azimuth angle are given in Equations (20) and (21), respectively,

(20) $$q\left(\theta_{k}\left(i\right)|\theta_{0:k-1}\left(i\right),y_{1:k}\right)=p\left(\theta_{k}\left(i\right)|\theta_{k-1}\left(i\right)，{\hat{\varphi }}_{k-1}\right)$$

(21) $$q\left(\varphi_{k}\left(i\right)|\varphi_{0:k-1}\left(i\right),y_{1:k}\right)=p\left(\varphi_{k}\left(i\right)|\varphi_{k-1}\left(i\right)，{\hat{\theta }}_{k-1}\right)$$

where ${\hat{\varphi }}_{k-1}$ and ${\hat{\theta }}_{k-1}$ are the estimated value of the state (pitch angle and the azimuth angle) in the previous moment, respectively. The particles $\left(\theta_{k}\left(i\right),{\hat{\varphi }}_{k-1}\left(i\right)\right)$ and $\left(\varphi_{k}\left(i\right),{\hat{\theta }}_{k-1}\left(i\right)\right)$ are obtained by sampling through the probability density represented in Equations (20) and (21). 

The essence of the modified PF algorithm is to construct new particle samples by exploiting the estimated angle in the previous moment, which is equivalent to the expansion of the particle number. Since the particle samples of the pitch angle and the azimuth angle at the *k* moment are relevant to the samples at the *k* − 1 moment and irrelevant to the estimated value at the *k*−1 moment, Equations (20) and (21) satisfy

(22) $$p\left(\theta_{k}\left(i\right)|\theta_{k-1}\left(i\right),{\hat{\varphi }}_{k-1}\right)=p\left(\theta_{k}\left(i\right)|\theta_{k-1}\left(i\right)\right)$$

(23) $$p\left(\varphi_{k}\left(i\right)|\varphi_{k-1}\left(i\right),{\hat{\theta }}_{k-1}\right)=p\left(\varphi_{k}\left(i\right)|\varphi_{k-1}\left(i\right)\right)$$

Substitute Equations (22) and (23) into Equation (17)

(24) $$w_{k\theta }^{*}\left(i\right)=w_{\left(k-1\right)\theta }^{*}\left(i\right)p\left(Y_{k}|\theta_{k}\left(i\right),{\hat{\varphi }}_{k-1}\left(i\right)\right)$$

(25) $$w_{k\varphi }^{*}\left(i\right)=w_{\left(k-1\right)\varphi }^{*}\left(i\right)p\left(Y_{k}|\varphi_{k}\left(i\right),{\hat{\theta }}_{k-1}\left(i\right)\right)$$

After normalization, the weights are represented by:

(26) $$w_{k\theta }\left(i\right)=\frac{w_{k\theta }^{*}\left(i\right)}{\sum_{i=1}^{N}w_{k\theta }^{*}\left(i\right)}\;$$

(27) $$\;w_{k\varphi }\left(i\right)=\frac{w_{k\varphi }^{*}\left(i\right)}{\sum_{i=1}^{N}w_{k\varphi }^{*}\left(i\right)}$$

Hence, the estimated values of the impinging angle are

(28) $$\varphi_{k}=\sum_{i=1}^{N}w_{k\varphi }\left(i\right)\varphi_{k}\left(i\right)$$

(29) $$\theta_{k}=\sum_{i=1}^{N}w_{k\theta }\left(i\right)\theta_{k}\left(i\right)$$

### 3.3. DOA Tracking and Estimation Algorithm Based on Modified Particle Filtering

In the modified PF algorithm, the weights selection of the particles affects the filtering performance. When the particles are near the real state, the weights of the likehood function of the particles are large and *vice versa*. The particles with large weights are duplicated in the resampling process. Hence, from Equation (16), it is known that the selection of the likehood function is crucial for the modified PF algorithm. 

The spectrum estimation of the MUSIC algorithm [8] is represented by:

(30) $$P_{MUSIC}(\theta ,\varphi )=\frac{1}{a^{H}\left(\theta ,\varphi \right)GG^{H}a\left(\theta ,\varphi \right)}$$

Then, Equation (30) is used to improve the likehood observation function and the sampling particles are evaluated by

(31) $$\hat{p}\left(Y_{k}|x_{k}\left(i\right)\right)=\frac{1}{a^{H}\left(x_{k}\left(i\right)\right)GG^{H}a\left(x_{k}\left(i\right)\right)}$$

In [19], the exponential weights factor *r* was added to the likehood function, which increased the distinctiveness of the weights and solved the problem of invalid sampling of the particles, due to the flat of the likehood function in the low SNR. Hence, the likehood function is rewritten as

(32) $$p\left(Y_{k}|x_{k}\left(i\right)\right)={\left\{\hat{p}\left(Y_{k}|x_{k}\left(i\right)\right)/\max_{x_{k}^{i}}\hat{p}\left(Y_{k}|x_{k}\left(i\right)\right)\right\}}^{r}$$

where *r* ∈ *R*^+^, and its value is determined by experiments. 

In summary, the steps of the DOA tracking and estimation algorithm based on MPF and MUSIC (MPF-MUSIC) are summarized as follows: 

Step 1: Initiation. The initial angles are estimated by random method. That is, the initial orientation is unknown and the angles are sampled from the uniform distribution with the number N and the weight 1/*N* of the particles:

$$\theta_{0}\left(i\right)∼U\left[0,\pi /2\right],\varphi_{0}\left(i\right)∼U\left[0,\pi \right],{\overset{\cdot }{\varphi }}_{0}\left(i\right),{\overset{\cdot }{\theta }}_{0}\left(i\right)∼N\left(\mu_{0},\varepsilon_{0}^{2}\right)\;i=1,\cdot \cdot \cdot ,N$$

where *U*[*e*_1_,*e*_2_] indicates the uniform distribution in the interval [*e*_1_,*e*_2_] and $\text{N}\left(\mu_{0},\varepsilon_{0}^{2}\right)$ indicates Gaussian distribution with mean $\mu_{0}$ and variance $\varepsilon_{0}^{2}$.

Step 2: At the k moment, the receiving matrix is calculated according to Equation (10) and the noise subspace is obtained.

Step 3: $\left(\theta_{k}\left(i\right),{\hat{\varphi }}_{k-1}\left(i\right)\right)$ and $\left(\varphi_{k}\left(i\right),{\hat{\theta }}_{k-1}\left(i\right)\right)$ are sampled according to Equations (20) and (21), respectively; the likehood functions $p\left(Y_{k}|\varphi_{k}\left(i\right),{\hat{\theta }}_{k-1}\left(i\right)\right)$ and $p\left(Y_{k}|\theta_{k}\left(i\right),{\hat{\varphi }}_{k-1}\left(i\right)\right)$ are calculated according to Equation (32); and the weights of the particles $w_{k\theta }^{*}\left(i\right)$ and $w_{k\varphi }^{*}\left(i\right)$ are calculated according to Equations (24) and (25), respectively. 

Step 4: The normalized weights are calculated according to Equations (26) and (27). 

Step 5: The estimated values of the state are calculated according to Equations (28) and (29).

Step 6: Let *k* = *k* + 1 and repeat steps from 2 to 6. If *k* > *T* where *T* is the observation length, the algorithm stops.

The differences of the steps of the DOA tracking and estimation algorithm based on PF and PF-MUSIC are merely in Step 3 to Step 5 of which in MPF-MUSIC, that is:

Step 3*: (*θ_k_* (*i*), *φ_k_* (*i*)) is sampled according to Equation (16), the likehood function $p\left(Y_{k}|\left(\theta_{k}\left(i\right),\varphi_{k}\left(i\right)\right)\right)$ is calculated according to Equation (32) and the weights of the particles $w_{k}^{*}\left(i\right)$ are calculated according to Equation (17).

Step 4*: The normalized weights *w_k_* (*i*) are calculated according to Equation (18) and resampled. 

Step 5*: The estimated values of the state are calculated according to Equation (19).

## 4. Simulation and Analysis 

### Single Array Element Experiment

Experiment 1: dynamic DOA tracking and estimation experiment based on PF and the corresponding modified algorithms

**Figure 1:**
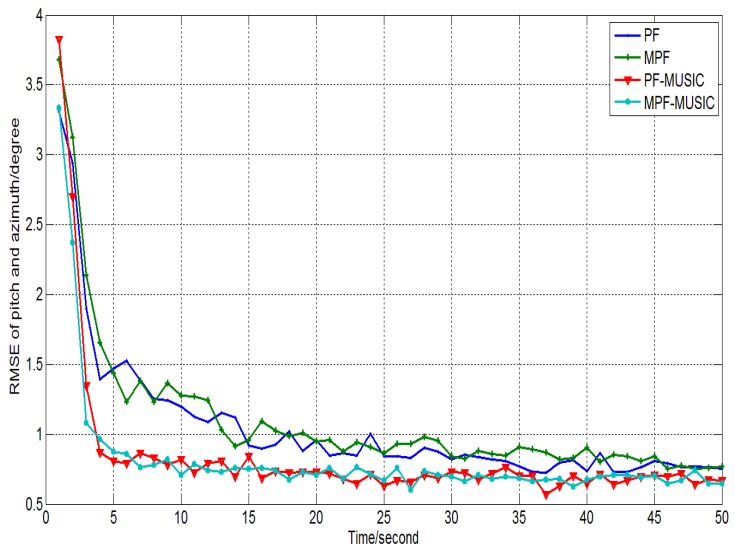
The RMSEs of the dynamic DOA tracking for the PF and the corresponding modified algorithms in different time.

Experimental conditions: single array element, *M* = 1, water is the propagation medium, the initial angle of the moving acoustic source is (30°, 20°) the moving interval is 1°, the snapshot is 256. In PF algorithm, the particle number is *N* = 200. In the modified PF algorithm, the particle number is *N* = 100, $\mu_{0}=\pi /180,\varepsilon_{0}^{2}=5\times 10^{-4}$, *r* = 13 which is determined by experiment. Let SNR = 5dB, observing the tracking deviation of the pitch angle and the azimuth angle, the experimental results is shown in Figure 1.

As indicated in Figure 1, each algorithm accomplishes the angle tracking when the observation time is longer than 5 s. In the initial stage, the estimation deviation is large and the range of the deviation is within 4°. Since the initial of the azimuth angle is selected randomly from the uniform distribution, the deviation of the PF algorithm is large at the initial moment and tends to stabilize after seconds, as shown in Figure 1. After a period of time estimation and correction, the fluctuation range of the tracking deviation of each algorithm decreases and tends to stabilize. Usually, the samples of the initial moment can be determined by DOA estimation algorithm, which decreases the fluctuation error of the algorithm in the initial stage. Moreover, the dynamic RMSE of the MPF-MUSIC algorithm seems identical with that of the PF-MUSIC algorithm in different time, but the probability of convergence (PROC) is better than that of the PF-MUSIC algorithm, which will be discussed in Experiment 3.

Experiment 2: the experiment of the weight factor affecting the DOA tracking deviation based on standard PF filtering.

Experimental conditions: the values of the SNR are −5 dB, 0 dB and 5 dB, respectively. The simulation times of Monte Carlo experiments are 100. Other experimental parameters are same the as Experiment 1.

**Figure 2:**
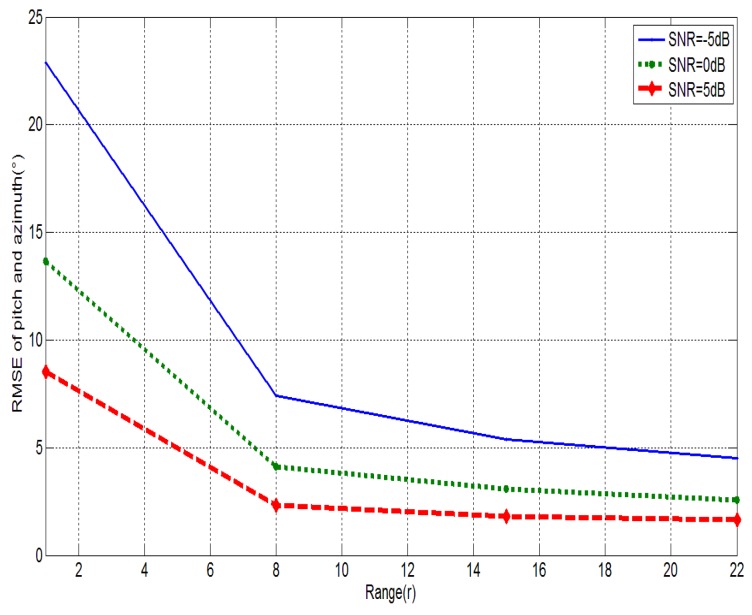
The RMSE sunder different exponential factors.

The Monte Carlo experiments are simulated independently for each exponential factor with the value of *r* changing from 1 to 22. The evaluation way of the estimation performance is the joint RMSE represented by:

(33) $$\text{RMSE=}\frac{1}{T}\sum_{j=1}^{T}\langle \sqrt{\frac{1}{100}\sum_{i=1}^{100}\left[{\left({\hat{\theta }}_{ij}-\theta_{ij}\right)}^{2}+{\left({\hat{\varphi }}_{ij}-\varphi_{ij}\right)}^{2}\right]}\rangle$$

where ${\hat{\theta }}_{ij}$ and ${\hat{\varphi }}_{ij}$ are the estimation values of the pitch angle and the azimuth angle in the *i*th simulation of the *j*th step. The simulation results are shown in Figure 2.

As indicated in Figure 2, the value of the RMSE decreases with the increase of *r*. When the value of *r* is large than 13, the tracking error of the algorithm tends to stabilize. Hence, *r* = 13 is selected in the simulation. Similarly, *r* = 6 is selected in the PF-MUSIC algorithm and MPF-MUSIC algorithm. 

Experiment 3: the PROC experiment of the PF tracking algorithm under different SNR.

The definition of convergence:

(34) $${ϒ}_{ki}\text{=}\{\begin{array}{c} 1\;\\ 0 \end{array}\;\begin{array}{c} \left|{\hat{\theta }}_{i}-\theta_{i}\right|+\left|{\hat{\varphi }}_{i}-\varphi_{i}\right|<\zeta \\ others \end{array}$$

where ${\hat{\theta }}_{i}$ and ${\hat{\varphi }}_{i}$ are the estimated value of the pitch angle and the azimuth angle in the *i*th simulation at the *k* moment, *ζ* is the threshold of the angle error and equal to 4 in the experiments. If the error is smaller than *ζ*, the estimated value is regarded as a successful estimation. 

The calculation formula of the probability of convergence is:

(35) $$PROC=\sum_{i=0}^{MC}{ϒ}_{ki}/MC$$

where *MC* is the times of the Monte Carlo experiments.

**Figure 3:**
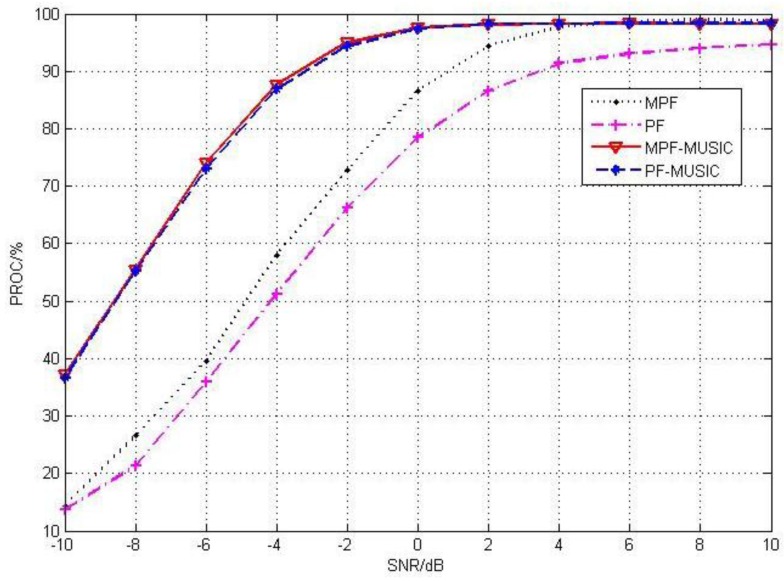
PROC with different SNRs.

Experimental conditions: the range of the SNR is from −10 dB to 10 dB and the step size is 2. Other experimental parameters are the same as those in Experiment 1. The simulation times of Monte Carlo experiments are 500, the evaluation way of the performance is according to Equation (35) and the simulation results are shown in Figure 3.

As indicated in Figure 3, the MPF algorithm has higher PROC than that of PF algorithm and achieves 99% when the SNR is equal to 10 dB. The PROC of the MPF-MUSIC algorithm is comparable with that of the PF-MUSIC algorithm. Hence, the improvement of independent evaluation for the pitch angle and the azimuth angle increases the PROC of the angle, which is significant in practical engineering. 

Experiment 4: the DOA tracking performance experiments of PF tracking under different SNR. 

**Figure 4:**
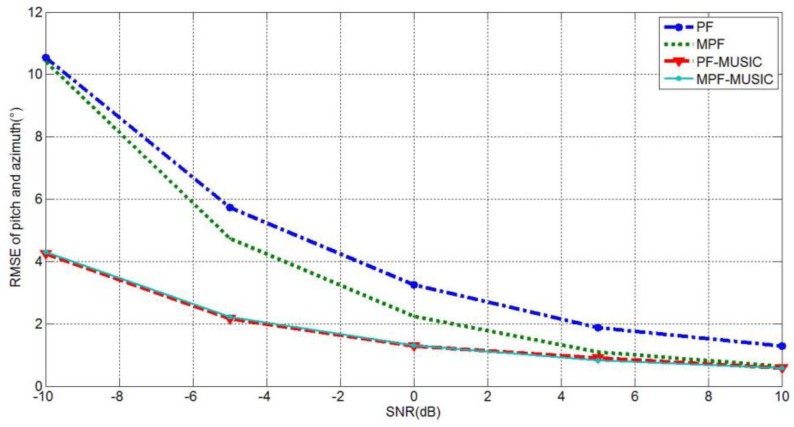
The RMSEs under different SNRs.

Experimental conditions: the range of the SNR is from −10 dB to 10 dB and the step size is 5. Other experimental parameters are the same as those in Experiment 1. The simulation times of Monte Carlo experiments are 100, the evaluation way of the performance is according to Equation (33) and the simulation results are shown in Figure 4.

As indicated in Figure 4, the joint RMSE of each tracking algorithm decreases with the increase of the SNR. Compared with the standard PF algorithm, the MPF algorithm proposed in the article has better estimation performance. Among the different PF algorithms, the tracking error of the PF-MUSIC algorithm is smaller than that of the PF algorithm. When the SNR is more than −5 dB, the tracking error is within 2°. Moreover, the RMSEs of the MPF-MUSIC algorithm under different SNRs are almost identical with those of the PF-MUSIC algorithm, but the PROC is also better than that of the PF-MUSIC algorithm, as discussed above.

## 5. Conclusions

This article studies the target tracking algorithm based on acoustic vector sensor array and proposes a modified particle filtering algorithm based on sequential importance sampling. The proposed algorithm solves the problems of large deviation of either pitch angle or azimuth angle leading to the other angle that cannot be duplicated and propagated through resampling in the two dimensional angle estimation. Furthermore, the MUSIC spectrum estimation algorithms are introduced as the observation likehood function of the modified particle filtering algorithm to improve the tracking performance and the combination of the modified particle filtering algorithm and MPF-MUSIC algorithm is proposed. As indicated in the experiments, the performance of MPF algorithm is better than that of PF algorithm, which has higher PROC. Furthermore, the performance of PF-MUSIC and MPF-MUSIC algorithms are better than that of PF and MPF algorithms in the aspects of RMSE, respectively. However, the MPF-MUSIC algorithm achieves comparable RMSEs and PROC with those of the PF-MUSIC algorithm. 
